# Philosophical Skepticism Concerning the Neutral Theory or Randomness: Misplaced or Misconceived? A Reply to Madison, “Stochasticity and Randomness in Community Assembly: Real or As-If?”

**DOI:** 10.1128/mSystems.01014-21

**Published:** 2021-09-07

**Authors:** Zhanshan (Sam) Ma

**Affiliations:** a Computational Biology and Medical Ecology Lab, State Key Laboratory of Genetic Resources and Evolution, Kunming Institute of Zoology, Chinese Academy of Sciences, Kunming, China; b Center for Excellence in Animal Evolution and Genetics, Chinese Academy of Sciences, Kunming, China

## REPLY

I appreciate a recent communication from Dr. Joseph Madison (1), titled “Stochasticity and randomness in community assembly: real or as-if?,” regarding my recent article (2), titled “Cross-scale analyses of animal and human gut microbiome assemblies from metacommunity to global landscape,” both published in *mSystems*.

I would like to first point out a couple of errors and/or misrepresentations of my conclusions in the first paragraph of Dr. J. Madison’s letter, from which I quote here: 'In a recent paper by Ma ([Z. Ma, mSystems 6:e00633-21, 2021]), results of a modified multisite neutral model analysis of community assembly inform a provocative claim that stochasticity correlates with a phylogenetic timeline. Two related results are that humans exhibit complete stochastic neutrality in microbial community assembly and that these results are of significance in understanding assembly processes. Consequently, Ma says: “[I]f the stochastic (random) forces are indeed significant in maintaining microbiome structures, the design of human interventions to maintain or restore microbiome structures … cannot ignore this important, innate aspect of the animal/human microbiome.” '

(i) Modification to the so-termed “multisite neutral model” seems to be exaggerated since no modification was made to the model in my paper.

(ii) The only occurrence of “stochastic neutrality” in my paper is quoted here: “We postulate that it should be the human experiences from agricultural/industrial activities (e.g., diet effects) and frequent social/familial contacts that are responsible for the dramatically rising stochastic neutrality in human gut microbiomes.”

(iii) The only occurrences of “complete” in my paper are “…to complete the computation had we applied the procedure to all 4,903 samples of the AGM data sets” and “In fact, completely checking each SAD-MSN data set with the Hammal et al. [3] approach will be hundreds (thousands) of times of computational load increases.”

I have also checked some synonyms of “complete” such as “totally/fully” in my paper but still failed to find the interpretation Dr. J. Madison had paraphrased from my paper. Of course, the combinations of the above-searched keywords with “stochastic neutrality” were not detected in my paper either. Since it seems hardly possible to examine every combination of paraphrasing my work to support Dr. J. Madison’s characterizations of my conclusions and/or claims, I would like to quote the last sentences in the “Importance” section of my article, to present an example of my writing style, which I believe can be characterized as cautious and was consistently followed in my paper: “The analyses were implemented by fitting the multisite neutral model and further augmented by checking false-positive and false-negative errors, respectively. It appears that there is a turning (tipping) point in the neutrality level from animal to human microbiomes.”

Given the above facts, I cannot help thinking whether or not Dr. J. Madison failed to make clear distinctions between postulation and conclusion, between correlation and causality, when characterizing my conclusions and/or claims and consequently leading to obvious exaggeration and/or misrepresentation. In addition, I have little objections to the direct quotes Dr. J. Madison excerpted from my paper, and I stand by those statements, although I am not very comfortable with the place those quotes were placed, the place to set a target for his follow-up comments and criticism. Due to the unfortunate misrepresentation of my conclusions and particularly the context setting, the rest of the paragraphs (after the first paragraph) of Dr. J. Madison’s letter actually have little relevance to my work. Nevertheless, I would still like to make the following comments. In my humble opinion, while the philosophy of biology is of critical importance for scientific research of biology, it should not replace scientific investigation of biological problems including the neutral theory. Understanding the problem and issues in the first place should still be a fundamental element of scientific research.

Nearly all of the issues in the rest of Dr. J. Madison’s letter after the first paragraph have been debated previously over the last 2 decades, and there are little novel insights in the letter in my opinion. For example, arguably the most serious criticism concerning the neutral theory of biodiversity has been the observations that both neutral and nonneutral models can generate similar or even identical SAD (species abidance distribution) patterns. While this is indeed an ecological reality, it is not without a measure to alleviate the issue. The following paragraph is excerpted from my article: “We realized that since the UNTB optimizes the fitting of the relative abundance to the predictions of the neutral model ([e.g., W. T. Sloan et al., Environ Microbiol 8:732–740, 2006; W. T. Sloan et al., Microb Ecol 53:443–445, 2007]), it might overestimate the true strength of neutral processes. Furthermore, some ecologists argue that even if empirical species abundance distributions (SADs) pass the neutrality test statistically, it does not guarantee that communities are truly neutral because nonneutral models can produce similar or even identical SAD patterns ([O. A. Hammal et al., PLoS Comput Biol 11:e1004134, 2015]). To deal with these issues, we adopted the following three tactics to reinforce the robustness of our study and more importantly deepen our understanding of the focal question raised in the manuscript title.” In my article, I applied Hammal et al.’s approach (3) to detect possible nonneutral processes in the SADs that have passed the neutral model simultaneously. The point I wish to make here is that Dr. J. Madison’s letter has obviously ignored such kinds of cautious efforts in applying the neutral theory, consequently slipping into philosophical skepticism.

I agree with Dr. J. Madison that the terms “stochastic” and “random” can represent different meanings. However, I would like to remind Dr. J. Madison that my paper is published in a journal of biology, not of philosophy or mathematics. Even in mathematics, I urge Dr. J. Madison to search Amazon.com and count how many books are titled “*Introduction to Random Processes*” and how many are titled “*Introduction to Stochastic Processes*”. I am neither arguing against the distinction between “random” and “stochastic,” nor do I fail to understand the meaning of randomness as an ecologist or as a student in a field using “*An Introduction to Kolmogorov Complexity and Its Applications*” (4) as a classic textbook. I just feel his argument may have been misplaced in the context of my paper since their meanings were self-evident if not interchangeable. If one wishes to use terminologies such as Kolmogorov set of infinite sequences, Bernoulii measure, and σ to discuss stochasticity and randomness in neutral theory, he or she should write a sufficiently detailed article to explain the context and justify one’s application of these concepts to the neutral theory, just as Vellend et al. and Wennekes et al. demonstrated (5, 6).

If you “do not know which of these conceptions is better” (1), would you please accommodate my being uncertain as expressed by “it appears that,” or “we postulate”? While allowing oneself to enjoy philosophical skepticism, what was wrong with my correlating phylogenetic timeline with stochasticity based on statistical tests? Should potential double standards be avoided?
